# CIDO, a community-based ontology for coronavirus disease knowledge and data integration, sharing, and analysis

**DOI:** 10.1038/s41597-020-0523-6

**Published:** 2020-06-12

**Authors:** Yongqun He, Hong Yu, Edison Ong, Yang Wang, Yingtong Liu, Anthony Huffman, Hsin-hui Huang, John Beverley, Junguk Hur, Xiaolin Yang, Luonan Chen, Gilbert S. Omenn, Brian Athey, Barry Smith

**Affiliations:** 1 University of Michigan Medical School, Ann Arbor, MI 48109 USA; 2 People’s Hospital of Guizhou Province, Guiyang, Guizhou 550002 China; 3 Guizhou University Medical College, Guiyang, Guizhou 550025 China; 4 National Yang-Ming University, Taipei, 112-21 Taiwan; 5 Northwestern University, Evanston, IL 60208 USA; 6 University of North Dakota School of Medicine and Health Sciences, Grand Forks, ND 58203 USA; 7 Institute of Basic Medical Sciences, Chinese Academy of Medical Sciences (CAMS) & School of Basic Medicine, Peking Union Medical College (PUMC), Beijing, China; 8 Key Laboratory of Systems Biology, Center for Excellence in Molecular Cell Science, Shanghai Institute of Biochemistry and Cell Biology, Chinese Academy of Sciences, Shanghai, 200031 China; 9 Center for Excellence in Animal Evolution and Genetics, Chinese Academy of Sciences, Kunming, 650223 China; 10 University at Buffalo, Buffalo, NY 14260 USA

**Keywords:** SARS-CoV-2, Viral infection, Computer science, Bioinformatics, Standardization

## Abstract

The Coronavirus Infectious Disease Ontology (CIDO) is a community-based ontology that supports coronavirus disease knowledge and data standardization, integration, sharing, and analysis.

Ontologies, as the term is used in informatics, are structured vocabularies comprised of human- and computer-interpretable terms and relations that represent entities and relationships. Within informatics fields, ontologies play an important role in knowledge and data standardization, representation, integration, sharing and analysis. They have also become a foundation of artificial intelligence (AI) research. In what follows, we outline the Coronavirus Infectious Disease Ontology (CIDO), which covers multiple areas in the domain of coronavirus diseases, including etiology, transmission, epidemiology, pathogenesis, diagnosis, prevention, and treatment. We emphasize CIDO development relevant to COVID-19.

Human coronaviruses have given rise to a series of major crises in global public health. Severe acute respiratory syndrome (SARS) emerged in China in November 2002, lasted for eight months and resulted in 8,098 confirmed human cases in 29 countries with 774 deaths (case-fatality rate: 9.6%)^1^. Approximately ten years later in June 2012, the Middle East Respiratory Syndrome (MERS), another highly pathogenic coronavirus disease, was identified in Saudi Arabia. The MERS outbreak has caused 2,260 cases in 27 countries and 803 deaths (35.5%)^2^. More recently, the World Health Organization (WHO) declared the Coronavirus Disease 2019 (COVID-19) outbreak as a pandemic on March 11, 2020, when there were 118,326 confirmed cases and 4,292 deaths. As of May 13, there have been over 4.4 million confirmed cases and over 295,000 deaths globally. Unfortunately, we still do not have available effective drugs and vaccines against these highly pathological coronaviruses.

Extensive studies have been conducted on coronaviruses, the results of many of which exist in publicly available data repositories such as GEO^3^. Publications concerning COVID-19 have exploded in recent months, and new clinical trials have been and are being conducted to develop drugs and vaccines against COVID-19, 1,430 of which have been registered in ClinicalTrials.gov as of May 13, 2020. As of May 13, 2020, a PubMed search of “SARS”, “MERS”, and “SARS-CoV-2 OR COVID-19” resulted in 12,993, 4,493 and 11,813 publications, respectively. A coordinated study of all such results would likely help with understanding and developing treatments for COVID-19. This coordinated study requires the integration of the large and exponentially growing data and research concerning COVID-19 to better understand its etiology, transmission, and pathogenesis mechanism. Moreover, we must be able to translate that understanding into rapid development of patient stratification methods leveraging precision medicine, therapeutic drugs, and preventive vaccines. However, there are two bottlenecks to achieving these tasks:

First, the characteristic five V’s of our Big Data^4^ era lead to disintegrated and non-interoperable data and knowledge. The amount of data (volume), speed at which it is produced (velocity), range of its sources (variety), quality and accuracy (veracity), and assessment of utility (value), result in large, complex, multidimensional, and diverse datasets. Disintegrated and non-interoperable data cannot be interpreted by computers and this inhibits computer-assisted reasoning, which is the essence of artificial intelligence. Consequently, our knowledge – data and information that embodies awareness and understanding – of domains represented by various datasets is seriously hindered. This is a familiar problem for biomedical research in general, which relies heavily on data acquisition, and for coronavirus research in particular, given the global challenge we currently face. The second bottleneck is the lack of bioinformatics tools that can efficiently and robustly integrate and analyze heterogeneous data and knowledge. This is likely a major stumbling block that is slowing the discovery of effective measures against coronaviruses even despite extensive effort across the globe.

A critical key to data/information/knowledge disintegration and big data analysis is ontologies. Ontologies are widely used in biomedical data and metadata standardization, and robustly support data integration, sharing, reproducibility, and computer-assisted data analysis. Ontologies are also regarded as the foundation of knowledge representation and reasoning (KR², KR&R), a major field of artificial intelligence. An important biomedical example is the Gene Ontology (GO)^5^, which was originally developed in the late 1990s by researchers studying the genomes of three model organisms: fruit fly, mouse, and yeast (*Saccharomyces cerevisiae*), but later extended to provide terms and relations used to annotate genes from humans, plants, animals, and microbes. GO includes three branches, for *cellular components*, *molecular functions*, and *biological processes*, forming a controlled vocabulary that can be used to represent attributes of gene products in a species-neutral way. Many GO-based tools and algorithms have been developed^6^. Since publication in 2000, the original GO paper^5^ has been cited some 25,000 times. GO makes possible consistent and reproducible annotations and analyses of genes and genomes from and across different organisms.

The success of GO inspired the development of hundreds of biomedical ontologies over the past two decades^7^. Included among those ontologies are many relevant to COVID-19: the Disease Ontology (DOID)^8^ classifies 18,000 human diseases now including COVID-19; the Human Phenotype Ontology (HPO)^9^ defines 26,000 human phenotypes; the Chemical Entities of Biological Interest ontology (ChEBI)^10^ includes 135,000 chemical entities; the Ontology for General Medical Sciences (OGMS) includes 200 terms for general medical classification; and the Ontology for Biomedical Investigations (OBI)^11^ includes nearly 4,000 terms related to all aspects of biomedical investigations. All these ontologies can be applied to the study of coronavirus diseases. Of most relevance to us here is the Infectious Disease Ontology (IDO)^12^, which defines 550 terms relating to infectious diseases in general and provides a basis for more specific IDO ontologies, for flu, malaria, brucellosis, and other diseases.

Ontology openness and interoperability are critical for data sharing and integration. The FAIR Guiding Principles propose that all research data should be Findable, Accessible, Interoperable and Reusable (FAIR) for both machine and human users^13^. “Interoperability” is the basis of the four FAIRness principles, which see ontology interoperability as the foundation of data/information/knowledge interoperability. With hundreds of ontologies developed, many ontologies overlap each other but, unfortunately, are not interoperable. Many ontologies, through lack of interoperability with other, more widely used ontologies, form silos and thereby fail to support integrative research. To foster interoperability, the Open Biomedical and Biological Ontologies (OBO) Foundry was initiated in 2007 by ontology developers who agreed to adopt a set of principles – including the commitment to collaboration and openness, use of definitions in both human- and computer-readable formats – specifying best practices in ontology development^14^. The OBO ontology library includes approximately 200 ontologies (including GO).

To meet the challenge of COVID-19, we recently initiated the development of CIDO, a community-driven open-source biomedical ontology in the area of coronavirus infectious disease (https://github.com/CIDO-ontology/cido). CIDO provides standardized human- and computer-interpretable annotation and representation of various coronavirus infectious diseases, including their etiology, transmission, epidemiology, pathogenesis, host-coronavirus interactions, diagnosis, prevention, and treatment. CIDO will be used as a state-of-the-art knowledge base for standard and logical representation of heterogeneous coronavirus knowledge. Having been accepted as an OBO library ontology, CIDO follows the OBO Foundry principles^14^, and uses an OBO-compatible extensible ontology development strategy^15^. To support data interoperability, CIDO reuses relevant coronavirus terms from existing reliable reference ontologies themselves aligning with OBO Foundry principles, and aligns these terms under the Basic Formal Ontology (BFO)^16^, an ISO/IEC standard 21838-2 (https://www.iso.org/standard/74572.html) top-level ontology. BFO is a realism-based ontology that covers all domains by providing highly general ontology classes such as material entity, process, role, site, and so forth. By using BFO as its upper-level architecture, CIDO is automatically interoperable and integrated with >300 other ontologies that also align with BFO. Currently, CIDO contains over 4,000 terms, imported from some 20 further ontologies such as ChEBI, Human Phenotype Ontology, Disease Ontology^8^, and the NCBI taxonomy ontology (NCBITaxon). Additionally, new CIDO-specific terms have been developed to meet the special needs arising in the research of COVID-19 and other coronavirus diseases.

Developing robust ontologies adequate for representing complex domains requires more than the simple construction of taxonomies. Taxonomies reflect important hierarchical relationships among class and subclass terms, represented using *is_a* relations. For example, instances of coronavirus are instances of viruses, which is to say coronavirus *is_a* virus. Extending beyond taxonomies, ontologies provide additional relations among entities within and across domains. Figure 1 illustrates a general design of how we can logically link ontology terms that may come from different branches of CIDO. The relations along the arrows in Fig. 1 are computer-understandable links between ontology classes. For example, we can define a logical axiom using the ‘caused by’ relation to link the COVID-19 disease process and SARS-CoV-2 virus:

**Fig. 1:**
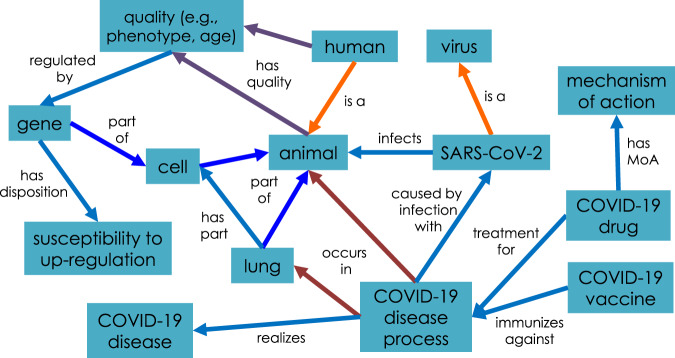
The design pattern of CIDO for logically representing and linking different components related to a coronavirus disease, e.g., COVID-19. The terms presented in the figure are generated in CIDO or imported by CIDO from other ontologies. To reduce complexity, the ontology sources of the terms are not labeled.

*COVID-19 disease process: ‘caused by infection with’ some SARS-CoV-2*

Such an axiom defines the causal relation between the COVID-19 disease process and the virus strain SARS-COV-2. More specifically, the development of the COVID-19 disease process (or realization of the COVID-19 disease disposition) in a patient is causally induced by an infection of the SARS-CoV-2 virus in the patient, involving viral invasion of and replication in host cells. In CIDO, diseases such as COVID-19 and pathogens such as SARS-CoV-2 have distinct hierarchies, with relations linking terms in these hierarchies. The inclusion of the relations (e.g., ‘caused by infection with’) in addition to *is_a* greatly expands expressiveness, reasoning capabilities, and expected inferences.

Figure 1 illustrates many other key relations. Particularly, COVID-19 occurs in the lung, and some genes in the cells of the lung would have the disposition of being susceptibly up- or down-regulated in the cells of SARS-CoV-2-infected lung. Such genes may function as gene markers and play important roles in pathogenesis. In addition, the infected patient will display different phenotypes after manifesting the disease, and such phenotypes may be associated with other patient attributes (e.g., biological sex, age) and the patient’s gene profile. CIDO thus provides semantically interoperable representations of host-coronavirus interaction mechanisms. Although Fig. 1 provides only a high-level overview of some CIDO resources, more details, such as specific signature genes in some cells of the lung that are susceptible to be up- or down-regulated in patients with COVID-19 will be added to the CIDO as new knowledge is acquired. Such systematic modeling and representation of the host-coronavirus interaction mechanisms would facilitate rational design of anti-coronavirus drugs and vaccines^17,18^.

In pursuit of that aim, CIDO can logically define relations between drugs and roles or mechanisms of action – distinct hierarchies in CIDO – and so support advanced analysis of potential drugs used to treat COVID-19, as well as the quick query of drugs having specific roles or mechanisms of action potentially useful as treatments. Such application of CIDO for ontology-based integration and analysis of anti-coronavirus drugs is shown in our recent preprint paper^17^. Using literature mining we identified 72 chemical drugs and 27 monoclonal or polyclonal antibodies that have anti-coronavirus effects in experimental studies *in vivo* or *in vitro*. Many of these drugs were mapped to three ontologies: Chemical Entities of Biological Interest ontology (ChEBI)^10^, National Drug File – Reference Terminology (NDF-RT)^19^, and the Drug Ontology (DrON)^20^. The subbranches of these ontologies that contain the mapped drugs and their related characteristics were extracted using the Ontofox tool^21^. Key information was identified by examining these subbranches. For example, based on their ChEBI annotations, many drug active ingredients are classified under the same chemical group: for example, chlorpromazine, dasatinib, terconazole, and chloroquine, all organochlorine compounds. Meanwhile, ChEBI classifies many drug chemicals having the same roles: chloroquine, conessine, lycorine, and mefloquine, all exhibit antimalarial activity. A ChEBI-based semantic similarity calculation method clustered 60 drugs into five major categories. The chemical information in ChEBI has also been imported to DrON. Developed by the U.S. Department of Veterans Affairs, Veterans Health Administration (VHA), NDF-RT organizes drugs by means of a formal representation of various drug characteristics such as mechanism of action (MoA), physiologic effect, and related diseases^19^. Using NDF-RT, we found that, of 35 drugs that have MoA annotations, 34 have MoAs of various inhibitors and antagonists. One shortcoming is that none of these ontologies covers all the needed information pertaining to our identified drugs. To study the anti-coronavirus drugs in a thorough manner we will need to identify and ontologically represent missing information of the sort that falls under the domain of the CIDO ontology. Thus, we plan to build logical relations linking drugs, coronaviruses, and the conditions under which the drugs work against the coronaviruses.

Another example of our ongoing work is the use of CIDO for the representation of vaccines against coronaviruses. We recently released another preprint paper on COVID-19 vaccine design using reverse vaccinology and machine learning^18^. Data pertaining to experimentally verified vaccine candidates in laboratory animal models have also been collected and annotated^18^. We will systematically annotate these vaccine candidates, including their formulations and host responses, and work with the Vaccine Ontology (VO) development team to model, represent, and analyze these vaccines (http://www.violinet.org/vaccineontology/). Moreover, CIDO can be used alongside VO to support literature mining of vaccine-associated gene-gene interactions^22^.

In future research, we will use ontology-based approaches to investigate relevant host-coronavirus interactions to support the fundamental understanding of the disease and protective immune system mechanisms, precision medicine research, and rational vaccine design^23,24^. More broadly, CIDO will provide community-based metadata standardization for interoperable and reproducible clinical and experimental studies, insofar as the coronavirus metadata ontology will be extracted from CIDO as a lightweight and relatively independent ontology to support data integration and knowledge discovery. We will use the standard to analyze clinical and basic research data and align the identified disease phenotype and transmission data with the underlying mechanisms introduced in other aims.

The extensive study of COVID-19 is generating new knowledge quickly. Given that our understanding of COVID-19 is changing rapidly, we need not only an ontology as wide in purview as CIDO, but also to be readily updated in a timely matter as more knowledge is generated about the disease, the virus, and the host response. To that end, we follow OBO Foundry guidelines for term requests and updating via issue tracking on the CIDO GitHub site (https://github.com/CIDO-ontology/cido). We welcome wide community participation in CIDO development and applications. CIDO is an open community; everyone is welcome. We are already collaborating with many groups and look forward to more collaborations with colleagues and people around the world. Starting with CIDO and its supported data integration, we expect that innovative computational and statistical algorithms and tools will be developed and applied to support basic studies of mechanisms, and translational applications such as predicting drugs and vaccines that hold promise for treating and preventing COVID-19.
